# The expanding role of m^6^A RNA modification in plant-virus dynamics: friend, foe, or both?

**DOI:** 10.1007/s44307-026-00100-3

**Published:** 2026-03-06

**Authors:** Jia-Hui Liu, Hao Yu, Cheng-Guo Duan

**Affiliations:** 1 State Key Laboratory of Plant Trait Design, CAS Center for Excellence in Molecular Plant Sciences, Chinese Academy of Sciences, Shanghai, 200032 China; 2 University of the Chinese Academy of Sciences, Beijing, 100049 China; 3 Department of Biological Sciences, National University of Singapore, Singapore, Singapore

**Keywords:** m^6^A, RNA modification, Plant virus, Plant-virus interaction, Epigenetics, Epitranscriptome

## Abstract

N^6^-methyladenosine (m^6^A), the most prevalent internal mRNA modification, regulates plant development and stress responses through modulating various mRNA metabolic processes and epigenetic effects. Although well studied in animals, its roles in plant–virus interactions have only recently begun to be elucidated. Multiple plant viruses carry m^6^A modifications on their RNAs, validated by MeRIP-seq, LC–MS/MS, and direct RNA sequencing. Viral RNAs acquire m^6^A through the recruitment or relocalization of host methyltransferase complexes, which is often mediated by viral proteins. Functionally, m^6^A can restrict infection by promoting viral RNA decay via YTH-domain readers and RNA surveillance pathways, or alternatively stabilize viral RNAs to enhance replication and systemic spread. In turn, viruses disrupt the functionality of host m^6^A machinery to promote infection. Moreover, viral infection reprograms host m^6^A homeostasis, altering methylation landscapes in immune and hormone pathways. These findings establish m^6^A as a dynamic epitranscriptomic switch in plant-virus interactions, with promising implications for antiviral strategies and crop improvement.

## Introduction

The epitranscriptome, defined by the reversible chemical modifications of RNA, has emerged as a key area of research in molecular biology. Among more than 170 known RNA modifications, N^6^-methyladenosine (m^6^A) is the most prevalent internal modification in eukaryotic messenger RNAs. Unlike irreversible nucleotide alterations, m^6^A functions as a dynamic and reversible mark, analogous to DNA and histone modifications, providing a rapid regulatory layer for post-transcriptional regulation.

The deposition of m^6^A is catalyzed by a conserved methyltransferase (writer) complex, which in mammals consists of METTL3, METTL14, WTAP, VIRMA, and RBM15/15B, and in plants of MTA, MTB, FIP37, VIRILIZER-like proteins (VIR), and HAKAI (Fu et al. 2014). The modification occurs at the consensus DRACH motif (D = A/G/U, R = A/G, H = A/C/U), enriched around stop codons and within 3′ untranslated regions. Dynamic removal of m^6^A is catalyzed by demethylase (erasers) such as FTO and ALKBH5 in animals and ALKBH9B and ALKBH10B in plants, ensuring reversible regulation. Functional interpretation is mediated by “readers”, primarily YTH domain-containing proteins, which selectively bind methylated transcripts to influence their fate.

Functionally, m^6^A impacts virtually every stage of the mRNA life cycle. In animals, m^6^A regulates splicing, nuclear export, mRNA stability, and translation efficiency. For instance, METTL3-mediated m^6^A is essential for embryonic development, stem cell differentiation, and neuronal plasticity (Quarto et al. 2025; Thombare et al. 2024; Yang et al. 2025). In plants, m^6^A contributes to embryogenesis, meristem maintenance, leaf morphogenesis, flowering time control, and stress adaptation (Arribas-Hernandez et al. 2018; Shao et al. 2025; Sharma et al. 2023; Shen and Yu 2025). Disruption of core m^6^A writers, such as MTA or FIP37, leads to embryonic lethality (Shen et al. 2016; Vespa et al. 2004; Zhong et al. 2008), underscoring the essential role of this modification in developmental programming. For instance, Arabidopsis MTA knockdown plants exhibit impaired growth under cold conditions (8 °C) compared to the wild type, accompanied by reduced abundance and translation of m^6^A-marked transcripts (Wang et al. 2023). The cold‐responsive gene *DGAT1* shows reduced translation in MTA RNAi plants, highlighting the functional relevance of m^6^A in stress adaptation. Similarly, m^6^A readers regulate gene expression through selective stabilization or sequestration of transcripts. ECT8, for example, sequesters the m^6^A-marked ABA receptor gene *PYL7* into stress granules to form phase separation condensates, thereby preventing its translation and attenuating ABA signaling (Wu et al. 2024).

Beyond endogenous functions, increasing evidence reveals that m^6^A also acts as a molecular interface in host interactions with exogenous factors, including host-virus interaction. In animal viruses, m^6^A exerts diverse and context-dependent roles, including modulating viral RNA stability, nuclear export, translation, and genome packaging (McFadden and Horner 2021; Williams et al. 2019). In RNA viruses, such as flaviviruses, retroviruses, and coronaviruses, dynamic methylation and the subsequent recognition by reader proteins influence replication efficiency and immune evasion (Williams et al. 2019). For example, m^6^A enhances Human Immunodeficiency Virus Type 1 (HIV-1) RNA export and protein production (Lichinchi et al. 2016a). In contrast, m^6^A supports replication and immune escape in Zika virus and influenza A virus (Lichinchi et al. 2016b; Wang et al. 2025). Conversely, in certain contexts, m^6^A restricts viral gene expression or promotes innate immune recognition. DNA viruses, including adenoviruses and herpesviruses, also exploit m^6^A to regulate transcription, splicing, and the balance between latent and lytic infection. Notably, Kaposi’s sarcoma-associated herpesvirus employs m^6^A to control latency-associated transcripts and reactivation. In parallel, m^6^A shapes host antiviral responses by modulating interferon-stimulated genes and innate immune sensors, with viruses often reprogramming the host m^6^A machinery to their advantage. Thus, m^6^A acts as a dynamic epitranscriptomic switch in animal viruses, simultaneously representing both a vulnerability and a weapon in infection, with potential implications for antiviral strategies.

A growing body of research indicates that plant viruses are similarly influenced by host-mediated m^6^A modification (He et al. 2023b; Secco et al. 2025). Infections with viruses such as *Cucumber mosaic virus* (CMV) alter m^6^A deposition on both host and viral RNAs (Liu et al. 2025), suggesting that m^6^A constitutes part of the molecular arms race between plants and viruses. In this review, we systematically summarize current knowledge of m^6^A function and mechanisms in plant–virus interactions and discuss key unresolved questions and future research directions.

## Validation of m^6^A modifications on plant viruses

For decades, it remained unclear whether plant viruses themselves carry this modification on their genomic or subgenomic RNAs. Since 2017, however, accumulating evidence has provided direct validation that plant viral RNAs are indeed subject to m^6^A modification (Table 1). The first biochemical evidence was reported by Martínez-Pérez et al., who used anti-m^6^A antibody–based immunoprecipitation of viral RNAs (MeRIP-qPCR) to demonstrate the enrichment of *Alfalfa mosaic virus* (AMV) RNAs (Martinez-Perez et al. 2017). In another study, Zhang et al. combined MeRIP-seq with a site-specific validation method to map high-confidence m^6^A peaks in *Wheat yellow mosaic virus* (WYMV) RNAs (Zhang et al. 2022). Similarly, Yue et al. reported that m^6^A peaks are enriched in discrete internal and 3′ terminal regions of *Plum pox virus* (PPV) and *Potato virus Y* (PVY) genomes in *Nicotiana benthamiana* (Yue et al. 2022). Further evidence was provided by He et al. (He et al. 2024, 2023a), who detected m^6^A in viral particles isolated from *Pepino mosaic virus* (PepMV)-infected tomato plants using both MeRIP-seq and dot blot analysis with anti-m^6^A antibodies.

**Table 1:** Viruses identified with m^6^A modifications

Virus	Host Plant	Identification Methods	Functions	Reference
*Alfalfa mosaic virus* (AMV)	*Arabidopsis thaliana*	MeRIP-seq, LC–MS/MS	Suppresses viral systemic infection	(Martinez-Perez et al. 2023, 2017)
*Barley yellow striate mosaic virus *(BYSMV)	*Hordeum vulgare* (Barley)	MeRIP-seq, m^6^A-IP qPCR	Promotes viral RNA stability and infection	(Zang et al. 2025)
*Pepino mosaic virus* (PepMV)	*Nicotiana benthamiana*; *Solanum lycopersicum*	MeRIP-seq, dot blot	Promotes viral RNA decay	(He et al. 2024, 2023a)
*Cucumber mosaic virus *(CMV)	*Arabidopsis thaliana*	MeRIP-seq, DRS, LC–MS/MS, dot blot	Promotes viral RNA decay	(Liu et al. 2025)
*Wheat yellow mosaic virus *(WYMV)	*Triticum aestivum*	MeRIP-seq, dot blot	Stabilizes viral RNA to promote infection	(Zhang et al. 2022)
*Sugarcane mosaic virus* (SCMV)	Maize	MeRIP-seq, DRS, SRAMP	Promote viral RNA decay	(Peng et al. 2026)
*Potato virus Y* (PVY)	*Nicotiana benthamiana*	MeRIP-seq, dot blot	Promote viral RNA decay	(Li et al., 2026)
*Bamboo mosaic virus* (BaMV)	*Dendrocalamus latiflorus* Munro	DRS	N/A	(Li et al. 2025)
*Rice stripe virus* (RSV) and *Rice black-streaked dwarf virus *(RBSDV)	*Oryza sativa*	MeRIP-seq	N/A	(Zhang et al. 2021a)

Most recently, an antibody-free nanopore direct RNA sequencing (DRS) approach has been employed to identify m^6^A in viral RNAs (Li et al. 2025; Liu et al. 2025; Peng et al. 2026). Liu et al. identified the presence of m^6^A in CMV genomic RNAs was validated through a combination of antibody-dependent MeRIP-seq, antibody-independent DRS, and LC–MS/MS analysis of viral particles. Notably, DRS enables single-base resolution mapping of m^6^A sites, and the majority of sites identified by DRS overlapped with MeRIP-seq peaks, thereby strengthening the reliability of the findings. A recent study demonstrated that *Sugarcane mosaic virus* (SCMV), a prevalent potyvirus infecting maize, undergoes m^6^A modification during infection (Peng et al. 2026). By integrating MeRIP-seq, DRS, and the sequence-based RNA adenosine methylation site predictor tool (SRAMP), the authors identified an m^6^A modification site at A6556 within the SCMV coding sequence. Furthermore, they showed that ZmMTA directs m^6^A deposition at A6556, thereby restricting SCMV infection. Consistently, a specific synonymous mutation (mutated A6556 to G) significantly enhanced SCMV infection. In another study, 122 potential m^6^A modification sites were identified in the genome of *Bamboo mosaic virus* (BaMV) through DRS approach (Li et al. 2025).

Together, these studies provide compelling and convergent evidence that m^6^A is an authentic epitranscriptomic modification of diverse plant viral RNAs.

## How viral genomic RNAs are recognized by host m^6^A machinery

In animal cells, m^6^A writer complexes are primarily nuclear. Many animal viruses, such as influenza virus and HIV, replicate in the nucleus, granting their RNAs direct access to the host’s m^6^A machinery that modifies host transcripts. Consequently, nuclear-replicating viral RNAs are more readily modified. Cytoplasmic RNA viruses, including flaviviruses such as Zika virus (ZIKV) and Dengue virus (DENV), were long thought to also acquire m^6^A through relocalization of the writer complex or its components to replication sites. However, this view has been challenged by recent evidence. For example, Baquero-Pérez et al. demonstrated that despite extensive investigation, Chikungunya virus (CHIKV) and DENV RNAs show no detectable m^6^A when analyzed with antibody-independent methods such as SELECT and nanopore sequencing (Baquero-Perez et al. 2024). Furthermore, depletion of m^6^A writer components had no measurable impact on infection, suggesting that m^6^A modification is not universal across cytoplasmic RNA viruses.

**Fig. 1:**
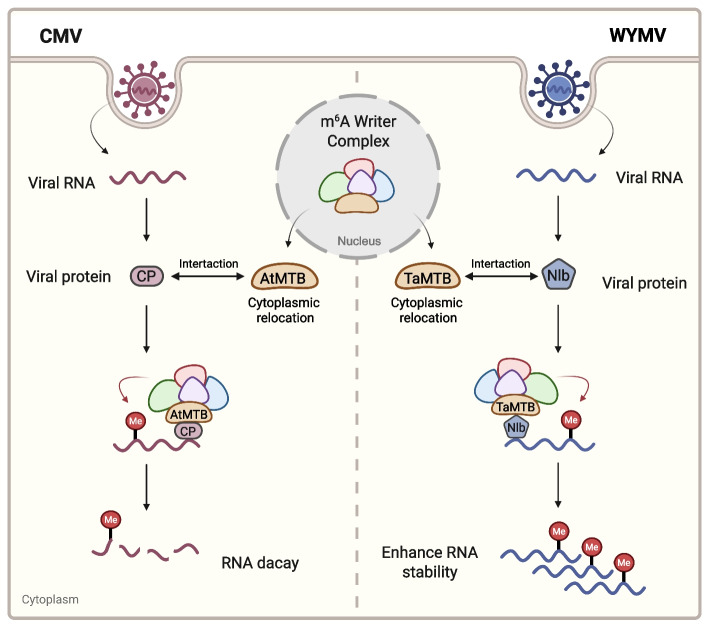
Recognition of viral genomic RNAs by the plant m^6^A writer complex. During CMV infection (left panel), the CMV-encoded CP protein directly interacts with AtMTB, a core component of the Arabidopsis m^6^A writer complex, to promote the nuclear-to-cytoplasmic translocation of the m^6^A writer complex, thereby facilitating m^6^A deposition on viral RNAs and subsequent RNA decay. Similarly, during WYMV infection (right panel), the WYMV-encoded NIb protein interacts with the *Triticum aestivum* m^6^A writer (TaMTB) to promote cytoplasmic relocation of the m^6^A writer complex and facilitate viral RNA recognition and m^6^A deposition, thereby stabilizing viral RNA and promoting infection. Image created in BioRender. Liu, J. (2026) https://BioRender.com/7m2p4da

In contrast, plant viruses, which predominantly replicate in the cytoplasm, appear to exploit distinct mechanisms to engage host m^6^A writers. Plant m^6^A writers can relocalize from the nucleus to viral replication complexes, often mediated by direct interactions with viral proteins. For example, in wheat, the writer TaMTB translocates to cytoplasmic replication sites through interaction with the WYMV NIb protein, methylating WYMV RNA at a defined site (Fig. 1). This modification enhances RNA stability and promotes infection, whereas mutation of the methylation site reduces viral stability and pathogenicity (Zhang et al. 2022). Similarly, in CMV (Fig. 1), the viral coat protein (CP) recruits m^6^A writers to the cytoplasm via direct interaction with the core writer MTB, thereby facilitating m^6^A deposition on viral RNAs (Liu et al. 2025). In addition, during SCMV infection, a co-localization of ZmMTA with SCMV genomic RNA in cytoplasmic aggregates (Peng et al. 2026), although the detailed ZmMTA relocation mechanism remains to be investigated. Taken together, these studies indicate that while nuclear replication provides direct access to host m^6^A writers, cytoplasmic viruses employ alternative strategies such as viral protein–mediated recruitment or relocalization of writer components. Importantly, not all viruses undergo m^6^A modification, as highlighted by CHIKV and DENV, underscoring the complexity and selectivity of host–virus epitranscriptomic interactions.

## Distinct modes of action of m^6^A in viral infection

### Control of viral RNA stability

Regulation of viral RNA stability represents a major mode of action for m^6^A, as demonstrated in multiple plant–virus systems. In CMV, hypermethylated viral RNAs are recognized by the reader protein ECT8, which directs RNA decay, possibly via the P-body RNA decay pathway, thereby restricting viral proliferation (Liu et al. 2025). Similarly, in PepMV infection of *Nicotiana benthamiana* (He et al. 2024), overexpression of the m^6^A writers MTA or HAKAI enhances viral RNA methylation and restricts infection, whereas deficiency in these factors reduces m^6^A levels and promotes viral accumulation. Mechanistically, m^6^A-marked viral RNAs are bound by cytoplasmic YTH-domain readers NbECT2A/2B/2C, which recruit nonsense-mediated decay (NMD) factors such as UPF3 and SMG7, leading to degradation of viral transcripts. Consistent with this pathway, PepMV RNA stability is significantly increased in NbECT2B-silenced plants, and silencing of NbUPF3 or NbSMG7 confers greater susceptibility to PepMV infection. In maize, the YTH domain-containing protein ZmECT23 recognizes viral m^6^A and destabilizes m^6^A-containing SCMV RNA through direct recruitment of the CCR4-NOT complex (Peng et al. 2026). More recently, Li et al. demonstrated that *Nicotiana benthamiana* writer NbMTA targets PVY genomic RNA for m^6^A deposition and promotes viral RNA degradation (Li et al. 2026).

By contrast, some viruses exploit m^6^A to enhance RNA stability and promote infection. In WYMV, the viral NIb protein recruits the wheat writer TaMTB to cytoplasmic replication sites, where site-specific m^6^A deposition stabilizes viral RNA and prevents degradation, thereby supporting viral accumulation (Zhang et al. 2022). A similar strategy is observed in the plant rhabdovirus *Barley yellow striate mosaic virus* (BYSMV), where the accessory gene *P6* mRNA is hypermethylated (Zang et al. 2025). Loss of m^6^A deposition reduces *P6* mRNA stability, resulting in a mutant virus with decreased infectivity. In barley, the m^6^A eraser HvALKBH1B acts as an antiviral factor by binding *P6* mRNA into cytoplasmic condensates via liquid–liquid phase separation (LLPS). The intrinsically disordered region of HvALKBH1B is essential for both LLPS and its antiviral function.

Together, these findings highlight the dual nature of m^6^A in viral RNA stability: while m^6^A often functions as an antiviral mark that targets viral RNAs for host surveillance and decay, some plant viruses hijack the host methylation machinery to stabilize their genomes and facilitate infection. This dynamic interplay underscores the complexity of m^6^A regulation in plant antiviral defense.

### Systemic movement and viral spread

Beyond RNA stability, m^6^A also regulates viral systemic movement. In AMV, the host eraser ALKBH9B removes m^6^A marks from viral RNAs, and this demethylation is required for efficient systemic infection. AMV infectivity depends on the interaction between the viral coat protein and ALKBH9B (Martinez-Perez et al. 2023, 2017). Excessive m^6^A deposition reduces phloem transport, thereby restricting long-distance spread (Zhang et al. 2021b). Thus, a balanced level of m^6^A is essential for efficient viral spread. Supporting this model, infection with PPV and PVY results in a global reduction of m^6^A levels in *N. benthamiana*, and down-regulation of ALKBH9 homologs significantly decreases PPV and PVY accumulation (Yue et al. 2022).

In addition to erasers, host reader proteins also regulate systemic infection. YTHDF proteins ECT2, ECT3, and ECT5 bind AMV RNAs in an m^6^A-dependent manner, functioning as restriction factors that suppress viral accumulation (Martinez-Perez et al. 2023). Interestingly, the regulation of AMV infectivity appears specific to ALKBH9B, since disruption of related homologs ALKBH9A or ALKBH9C does not affect viral infection (Martinez-Perez et al. 2021).

Collectively, these findings demonstrate that m^6^A modulates not only the stability of viral RNAs but also their capacity for systemic movement, with both host writers and erasers playing crucial yet context-dependent roles in shaping infection outcomes.

## Viral countermeasures against host m^6^A machinery

**Fig. 2:**
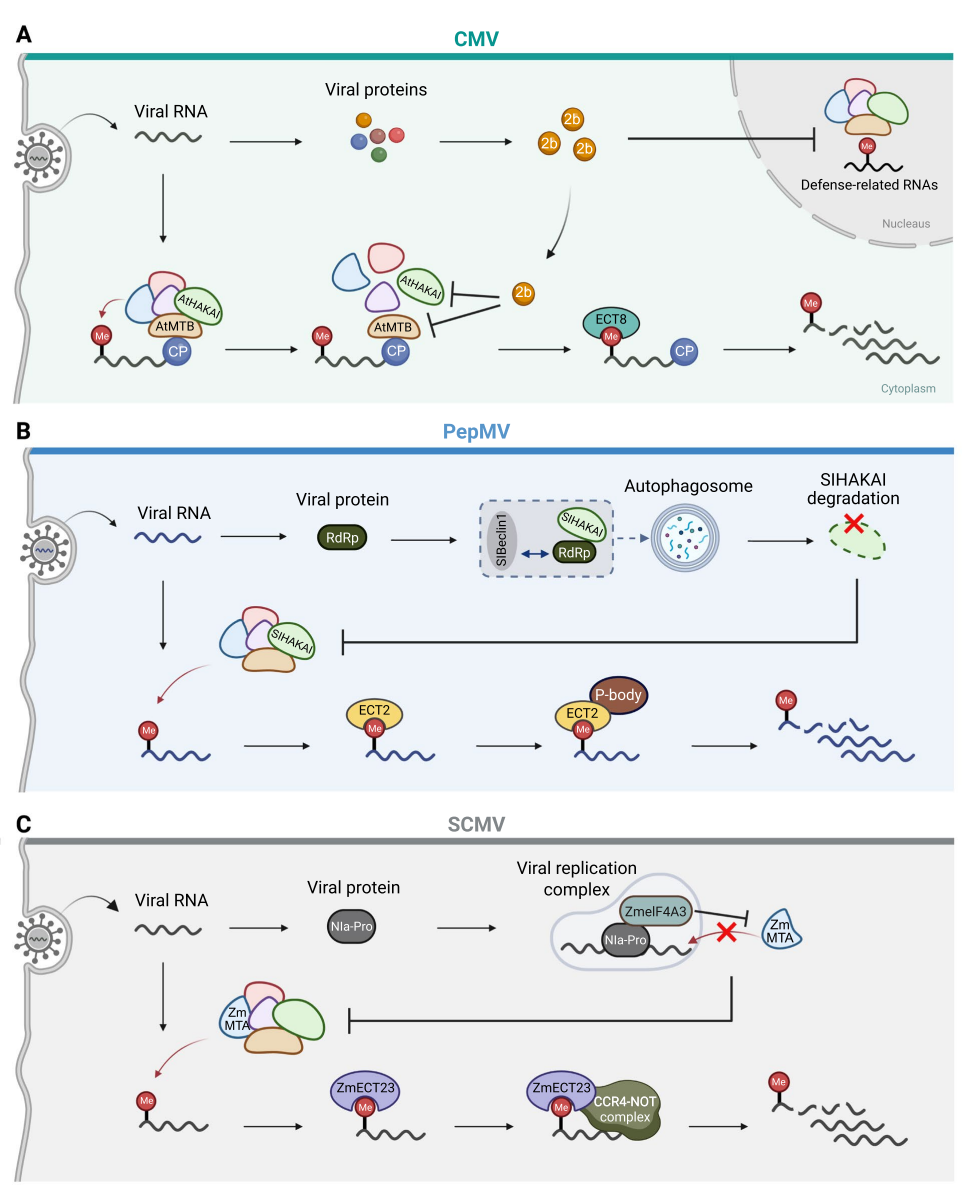
Mechanisms of viral antagonism in plant m^6^A-mediated antiviral defense. **A** During CMV infection, the CMV-encoded 2b protein competitively binds to the Arabidopsis m^6^A writer components MTB and HAKAI, thereby impairing the integrity and functionality of the m^6^A writer complex in both the nucleus and cytoplasm. This interaction suppresses m^6^A deposition on viral RNAs as well as host defense-related transcripts. **B** During PepMV infection, the viral RdRP protein interacts with the m^6^A writer SlHAKAI and the tomato autophagy-related protein SlBeclin1, leading to the formation of cytoplasmic interaction granules and facilitating autophagic degradation of SlHAKAI. Consequently, m^6^A deposition of viral RNAs is inhibited, resulting in enhanced viral RNA stability. **C** During SCMV infection, the maize m^6^A writer ZmMTA deposits m^6^A modification on viral RNA. These m^6^A marks are recognized by the reader protein ZmECT23, which recruits the CCR4-NOT complex to promote viral RNA degradation and thereby attenuating viral infection. In contrast, the viral protien NIa-Pro hijacks the host ZmeIF4A3 factor to the viral replication complex, inhibiting ZmMTA-mediated m^6^A deposition on viral RNA and promoting viral replication. Image created in BioRender. Liu, J. (2026) https://BioRender.com/wo81sl8

In the evolutionary arms race between plants and viruses, antiviral defense mechanisms are frequently counteracted by viral inhibitory strategies. Recent studies have demonstrated that viruses can actively manipulate m^6^A deposition on their genomes to attenuate host defense responses. Liu et al. (Liu et al. 2025) showed that during CMV infection, the viral 2b protein, a well-characterized suppressor of RNA silencing, suppresses m^6^A deposition on viral RNAs by impairing the function of the host m^6^A machinery (Fig. 2). Mechanistically, 2b competitively interacts with host m^6^A writer components HAKAI and MTB. Consequently, compared with wild-type plants, m^6^A-deficient plants exhibited increased susceptibility to 2b-deficient CMV but not to wild-type CMV. Furthermore, the 2b-deficient virus displayed significantly higher m^6^A levels than the wild-type virus. These findings reveal a mutually antagonistic interplay between m^6^A-mediated antiviral immunity and a viral countermeasure, positioning m^6^A dynamics as a critical battleground in the molecular arms race between plant hosts and viruses. Notably, 2b expression also exerts a profound inhibitory effect on global m^6^A levels in host plants.

In a separate study, He et al. reported that PepMV encodes a viral RNA-dependent RNA polymerase (RdRP) that promotes autophagy-dependent degradation of SlHAKAI, a core m^6^A writer component in *Solanum lycopersicum* (He et al. 2023a). Overexpression of *SlHAKAI* significantly restricted PepMV infection, underscoring its antiviral role. Mechanistically, PepMV RdRP directly interacts with SlHAKAI, inhibiting its protein accumulation via autophagic degradation. The autophagy-related protein SlBeclin1 has been shown to interact with the RdRPs of several RNA viruses and promotes their degradation (Li et al. 2018a). He et al. further revealed that the PepMV RdRP-mediated autophagic degradation of SlHAKAI requires SlBeclin1. RdRP, SlHAKAI, and SlBeclin1 form cytoplasmic interaction granules that facilitate autophagic degradation of SlHAKAI (Fig. 2). This process effectively reduces m^6^A methylation on viral RNAs, thereby enabling PepMV to evade m^6^A-mediated antiviral defenses.

More recently, Peng et al. revealed a sophisticated m^6^A-mediated antagonistic mechanism between maize and SCMV (Peng et al. 2026). On the one hand, the host m^6^A writer ZmMTA deposits m^6^A marks on SCMV genomic RNA, promoting viral RNA decay through ZmECT23-mediated recruitment of the host ZmCCR4-NOT deadenylation complex (Fig. 2). On the other hand, the SCMV nuclear inclusion protein a protease (NIa-Pro) hijacks the maize eukaryotic initiation factor 4A-III (ZmeIF4A3) into viral replication complexes, thereby blocking ZmMTA-mediated m^6^A deposition and preventing viral RNA from degradation (Fig. 2).

Collectively, these bidirectional regulatory mechanisms exemplify the dynamic interplay between host m^6^A-dependent antiviral defenses and viral strategies to subvert epitranscriptomic control.

## The impacts of viral infection on host m^6^A homeostasis

Beyond substantial modification of viral genomes by the host m^6^A machinery, viral infection also profoundly alters N^6^-methyladenosine (m^6^A) RNA methylation dynamics in plant hosts. Many plant viruses induce genome-wide remodeling of the host m^6^A methylome, leading to either hypermethylation or hypomethylation of host mRNAs depending on the virus–host context.

In *Nicotiana tabacum*, infection with *Tobacco mosaic virus* (TMV) results in decreased m^6^A levels in host mRNAs, which correlates with upregulation of the putative m^6^A eraser NbALKBH5 and reduced expression of m^6^A writer components (Li et al. 2018b). Similarly, in *N. benthamiana*, infection by PPV and PVY reduces m^6^A levels (Yue et al. 2022). By contrast, in rice plants infected with *Rice stripe virus* (RSV) or *Rice black-streaked dwarf virus* (RBSDV), host mRNA m^6^A levels are enriched (Zhang et al. 2021a). Notably, although both infections alter global m^6^A abundance, the specific m^6^A “peaks” (sites of modification) differ, indicating virus-specific reprogramming of the host epitranscriptome.

Distinct temporal and genotype-dependent responses have also been observed. In watermelon infected with *Cucumber green mottle mosaic virus* (CGMMV), susceptible plants showed increased m^6^A levels at 24 h post-infection, whereas resistant cultivars exhibited decreased levels by 48 h (He et al. 2021). In *Arabidopsis thaliana* infected with CMV, global m^6^A levels were markedly reduced, primarily due to inhibition of the host m^6^A writer complex by the viral 2b protein (Liu et al. 2025). Supporting this mechanism, transgenic expression of 2b alone led to a significant decrease in global m^6^A levels, while infection by viruses lacking 2b had no effect, underscoring the importance of specific viral proteins in perturbing host m^6^A homeostasis.

These alterations in m^6^A profoundly affect the expression of host genes involved in immunity, hormone signaling (e.g., salicylic acid, jasmonic acid), and stress responses, thereby reshaping antiviral defense networks. In rice infected with RSV or RBSDV, virus-induced m^6^A hypermethylation is enriched in transcripts associated with RNA silencing and hormone-mediated defense pathways, including antiviral genes such as *OsAGO18* and *OsSLRL1* (Zhang et al. 2021a). The extent of m^6^A methylation is closely correlated with changes in transcript abundance, and the effects of m^6^A modifications differ by gene region. Similar reprogramming occurs in CGMMV-infected watermelon, where susceptible plants exhibited early global m^6^A hypermethylation of stress- and defense-related transcripts, whereas resistant plants displayed later hypomethylation that preserved defense gene expression (He et al. 2021). In *A. thaliana*, CMV-induced m^6^A hypomethylation primarily resulted in the upregulation of immune genes, including *NPR3* and *CBP60a*, which are key regulators in salicylic acid-mediated defense (Liu et al. 2025). In *Nicotiana benthamiana*, infection by PVY induces dynamic alterations in global m^6^A levels, which are elevated during the early to middle stages of infection (5–10 days post-inoculation, dpi) but decline by 14 dpi (Li et al. 2026). PVY infection enhances the expression of the m^6^A-modified transcription factor NFYA3_0, which in turn activates transcription of the m^6^A writer NbMTA, thereby promoting m^6^A deposition on viral RNAs and accelerating viral RNA degradation. Li et al. demonstrated that BaMV infection induces a global increase of m^6^A levels in* D. latiflorus* accompanied with reduced proportion of full-length transcripts (Li et al. 2025). Epitranscriptome analysis showed increased m^6^A ratios in the chlorophyll synthesis genes *POR* and abscisic acid synthesis gene *NCED1*, which coupled with reduced transcriptional levels.

Collectively, these findings highlight that viral infection reprograms the host epitranscriptome and that dynamic m^6^A regulation acts as a fine-tuning mechanism in plant-virus interactions.

### Perspective

In plants, the dynamic interplay between m^6^A deposition and viral counterstrategies defines a key battleground of molecular plant immunity, offering potential leverage points for crop improvement and antiviral strategies. The emerging view of m^6^A as a pivotal regulator in plant–virus interactions highlights an additional epitranscriptomic layer of complexity in plant pathology. While initially characterized for its developmental roles, recent discoveries demonstrate that m^6^A marks on both host and viral RNAs actively shape the interaction between viral infection and host adaptation. Plant viruses, many of which replicate exclusively in the cytoplasm, rely on the capacity of the host m^6^A machinery to relocalize or be recruited to replication sites, thereby enabling the modification of viral genomes. These modifications can either restrict or promote infection depending on the specific virus-host context. For instance, in PepMV, hypermethylation of viral RNA triggers recognition by host YTH-domain readers and subsequent RNA decay, whereas in WYMV, recruitment of TaMTB by the viral NIb stabilizes viral transcripts and enhances accumulation. These findings underscore the dual role of m^6^A as both an antiviral defense strategy and a viral exploitation mechanism.

Equally important is the systemic dimension of m^6^A regulation. In AMV, balanced demethylation by ALKBH9B is required for efficient long-distance movement, illustrating that m^6^A does not act in a binary restrictive or permissive manner but instead fine-tunes infection dynamics to optimize viral spread. Moreover, virus-induced perturbations of host m^6^A homeostasis exert broad effects on transcriptome reprogramming. Distinct viruses remodel global methylation landscapes differently. CMV, TMV, and PPV induce hypomethylation of host mRNAs, while RSV and RBSDV increase methylation levels. These changes extend beyond individual transcripts to influence entire regulatory pathways, including hormone signaling and RNA silencing, thereby integrating m^6^A into the host’s broader stress and developmental networks.

The antagonistic interplay between host defenses and viral countermeasures adds further complexity. Viral proteins such as CMV 2b and PepMV RdRP directly target host m^6^A writers, either by competitive inhibition or by promoting autophagic degradation of core components. These strategies effectively suppress m^6^A deposition on viral RNAs, allowing viruses to evade restriction. In turn, plants have evolved erasers such as HvALKBH1B, which sequester and destabilize viral transcripts via liquid–liquid phase separation (Zang et al. 2025). The reciprocal adaptation of host and virus demonstrates that m^6^A is a central node in the molecular arms race of infection.

Looking forward, several key questions remain open. First, recent methodological studies have drawn attention to potential limitations in transcriptome-wide m^6^A detection. Notably, a recent report showed that false-positive m^6^A signals may occur not only in antibody-based m^6^A-RIP-seq but also in certain antibody-independent detection approaches, highlighting the necessity of appropriate negative controls and careful data interpretation (Pan et al. 2025). These observations suggest that current m^6^A mapping strategies may still be susceptible to technical artifacts, particularly when applied to complex biological samples. Looking forward, emerging technologies such as single-molecule and real-time RNA modification mapping are expected to improve the resolution and specificity of m^6^A detection. The continued development and integration of such approaches will be important for refining epitranscriptomic landscapes and for achieving more accurate and quantitative characterization of RNA modifications in diverse systems. Second, how are specific viral or host transcripts selectively targeted for m^6^A deposition or removal during infection? The identification of sequence and structural determinants will be essential to predict outcomes across different plant–virus systems. Third, what are the temporal dynamics of m^6^A reprogramming? Time-resolved mapping may reveal whether early infection stages favor antiviral methylation, while later stages are dominated by viral suppression mechanisms. Fourth, can the manipulation of m^6^A pathways be exploited for durable crop resistance? Overexpression of writer components or modulation of reader activity has shown promise in experimental systems, suggesting the potential for breeding or biotechnological strategies.

In conclusion, m^6^A represents a versatile regulatory hub that integrates viral replication strategies with host RNA metabolism. Its context-dependent roles, as both a host defense mechanism and a viral advantage, reflect its dynamic nature as an epitranscriptomic switch. A deeper mechanistic understanding of this modification in the plant-virus system holds promise for novel antiviral approaches and offers a paradigm for studying RNA modifications in complex host–pathogen interactions.

## Data Availability

No datasets were generated or analyzed during the current study.
